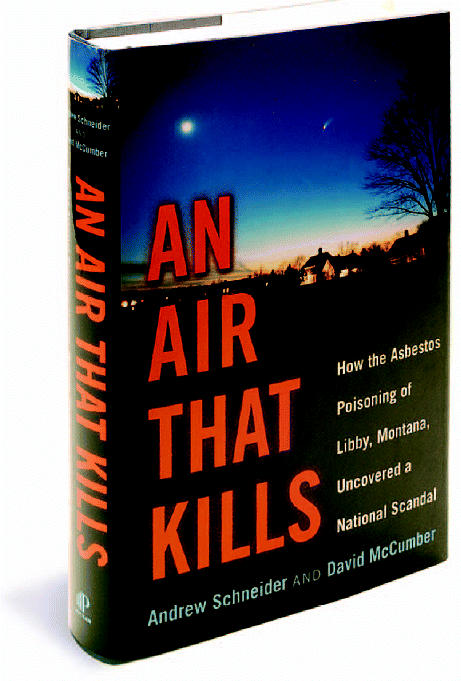# An Air that Kills: How the Asbestos Poisoning of Libby, Montana, Uncovered a National Scandal

**Published:** 2004-08

**Authors:** Howard M. Kipen

**Affiliations:** Howard M. Kipen is professor of Environmental and Occupational Medicine and director of the Division of Occupational Medicine at the UMDNJ–Robert Wood Johnson Medical School and its Environmental & Occupational Health Sciences Institute. He studies occupational respiratory disease and has consulted with the ATSDR on the Libby situation.

By Andrew Schneider and David McCumber

New York:G.P. Putnam’s Sons, 2004. 440 pp. ISBN: 0-399-15095-1, $25.95 cloth.

Asbestos has tragically affected communities throughout the world, largely through its effects on workers. This powerful history is presented with dramatic flair in the new investigative book by Andrew Schneider and David McCumber. *An Air That Kills* provides a compelling update on what should now be a closed chapter in occupational health in the United States. More remarkably, it also asserts the potential impact of the long-feared environmental health disaster arising from negligent use of asbestos. This book explores new territory in nonoccupational asbestos exposure.

The book records the history of some of the heroes of occupational medicine in the United States and also names some of the “black hats.” It also addresses risk and risk reduction related to nonoccupational exposure to asbestos. Here it lands on thin ice, and may even fall through it by omitting results of the Agency for Toxic Substances and Disease Registry (ATSDR) survey that could validate its thesis, as well as by making some extreme statements. Schneider and McCumber note that the federal government “did the biggest public health survey in its history. A third of the town got . . . the ‘death sentence.’” The data from the survey, not presented in this book although published in *EHP* (111:1753–1759), do not substantiate this statement. The nonrandom sample of 6,668 adults in Libby showed 17.8% with pleural abnormalities and < 1% with interstitial abnormalities. These findings are hard to reconcile with certain asbestos-related mortality of one-third of the town.

A wealth of investigative reporting exposes how environmental asbestos concerns were neglected by the Bush–Whitman Environmental Protection Agency (EPA) in the desire to get Wall Street going after September 11. The authors also describe vermiculite shipments to over 750 locations, 293 major users, and 45–73 expansion facilities throughout North America. Attic insulation, concrete, wallboard, roofing, crayons, and potting soil, as well as tremolite-contaminated talc, taconite ore, and Sierra foothill soil, contain contaminated vermiculite or other “naturally occurring asbestos.” No one has epidemiologically established the risk of mesothelioma from these environmental exposures. This book amply documents “an enormous hole in the safety net that people assume is out there in modern life” for toxic substance regulation and enforcement.

The allegation that Libby’s doctors rarely diagnosed asbestosis because doctors were high in the town’s caste system is telling but not documented. The story that W.R. Grace & Co. and others conspired to intimidate a doctor who was willing to diagnose asbestosis is convincing, as are other claims of Grace’s unethical behavior. The lack of chapter titles decreases the utility and approachability of this book. There are too many silly errors of fact, and it is frustrating to read a provocative statement characterizing the World Trade Center cleanup without a source citation. Such a source might likely be an interview rather than a report that could be reviewed by readers. This is not an academic book, but a popular and, to some extent, a muckraking book based on a lot of journalistic research.

While U.S. EPA scientists and investigators were struggling to document the Libby problem in the late 1990s, they uncovered reports from 1980 and 1982 that had already characterized the problem. But neither miners nor the public had been warned. The book alleges that 15 years before it took any concrete action, the U.S. EPA knew that asbestos was killing residents of Libby, stonewalled, and then in an about-face requested an investigation, eventually giving an award to the local administrators who had publicized both the situation and the U.S. EPA’s period of inaction. There are even implications that close ties between W.R. Grace CEO J. Peter Grace and the Reagan White House might have led to downplaying the earlier knowledge. The U.S. EPA had knowledge that could have begun a reduction in exposure, for miners and the town, 15–20 years sooner. W.R. Grace made denials until they declared bankruptcy.

Beyond the hyperbole and the melodrama of the individual tragedies, there is much new history and political insight to recommend this book. This is important material in the unfortunately still unfolding scientific and historical chapters on asbestos and disease.

## Figures and Tables

**Figure:**